# The enantiostylous floral polymorphism of *Barberetta aurea* (Haemodoraceae) facilitates wing pollination by syrphid flies

**DOI:** 10.1093/aob/mcad118

**Published:** 2023-08-26

**Authors:** Steven D Johnson, Jeremy J Midgley, Nicola Illing

**Affiliations:** Centre for Functional Biodiversity, University of KwaZulu-Natal, 3209 Pietermaritzburg, South Africa; Department of Biological Sciences, University of Cape Town, 7701 Cape Town, South Africa; Department of Molecular and Cell Biology, University of Cape Town, 7701 Cape Town, South Africa

**Keywords:** enantiostyly, floral adaptation, herkogamy, hoverflies, fly pollination, nectar guides, pollen dispersal, self-compatibility, Syrphidae

## Abstract

**Background and Aims:**

Sexual polymorphisms of flowers have traditionally been interpreted as devices that promote cross-pollination, but they may also represent adaptations for exploiting particular pollination niches in local environments. The cross-pollination function of enantiostyly, characterized by flowers having either left- or right-deflected styles, has been uncertain in some lineages, such as the Haemodoraceae, because the positioning of stamens and styles is not always completely reciprocal among morphs.

**Methods:**

We examined the floral biology of populations of the poorly known species *Barberetta aurea* (Haemodoraceae) across its native range in South Africa to establish the general features of its enanatiostylous reproductive system and the agents and mechanism of pollen transfer.

**Results:**

We confirmed that *B. aurea* has a system of dimorphic enantiostyly. Style morph ratios varied among populations sampled, but with an overall tendency to being equal. Crossing experiments demonstrated that *B. aurea* is fully self-compatible, that intra- and inter-morph crosses are equally fertile and that it is wholly dependent on pollinator visits for seed production. Pollination is mainly by syrphid flies that transfer the sticky pollen via their wings, which contact the anthers and stigma precisely as they hover during approach and feeding. The majority of syrphid fly visitors feed on a film of highly concentrated nectar situated at the base of ultraviolet-absorbent ‘nectar guides’. Because one of the three stamens is deflected in the same direction as the style, we predicted a high likelihood of intra-morph pollination, and this was corroborated by patterns of transfer of coloured dye particles in cage experiments involving syrphid flies.

**Conclusions:**

*Barbaretta aurea* exhibits dimorphic enantiostyly and, in contrast to most enantiostylous species, which are pollinated by bees, its flowers are specialized for pollination by syrphid flies. The lack of complete reciprocity of the enantiostylous arrangement of sexual organs facilitates both inter- and intra-morph pollen transfer on the wings of these flies.

## INTRODUCTION

The function of floral sexual polymorphisms has been a topic of interest to plant biologists since Darwin’s seminal work on the subject (*sensu*Darwin, 1877; Barrett, 2010). Most research has focused on distyly, a widespread polymorphism with multiple evolutionary origins that involves plants with either short styles and long stamens or with the reciprocal arrangement of long styles and short stamens (Ganders, 1979; Barrett, 1992). Distyly (and the less common tristylous polymorphism) is usually, but not always, linked to a heteromorphic incompatibility system that enforces disassortative (inter-morph) mating and maintains morph ratios (Barrett, 1992; Barrett and Cruzan, 1994). In contrast, enantiostyly is a relatively rare sexual polymorphism that involves styles deflected either to the left or right side of the mid plane of the flower, and these can co-occur on plants (monomorphic enantiostyly) or be segregated among plants (dimorphic enantiostyly) (Barrett, 2002; Jesson and Barrett, 2003). The latter type has been documented in three monocotyledonous families: Haemodoraceae (Ornduff and Dulberger, 1978; Jesson and Barrett, 2002*a*), Pontederiaceae (Jesson and Barrett, 2002c) and Tecophilaeaceae (Dulberger and Ornduff, 1980). In contrast to distyly, most enantiostylous species are monomorphic, and even in species that are dimorphic there is often a lack of complete reciprocity in organ positioning (Ornduff and Dulberger, 1978). There is also no strong evidence for heteromorphic incompatibility in dimorphic enantiostylous species (Ornduff and Dulberger, 1978; Jesson and Barrett, 2002*a*). Thus, in comparison to most other sexual polymorphisms, the function of enantiostyly and the maintenance of balanced morph ratios are not yet well understood.

Although the function of both distyly and enantiostyly has traditionally been attributed to selection for mechanisms that limit geitonogamous self-pollination (Richards, 1997; Jesson *et al.*, 2003), the evolution of these polymorphisms might also have been driven by selection for more efficient pollen transfer to stigmas (Barrett, 2002; Jesson and Barrett, 2005). One widely accepted scenario for the evolution of distyly is that in species with approach herkogamy (long style and short stamens), mutants with the reciprocal arrangement (‘reverse herkogamy’) would gain an immediate pollination advantage owing to their precision of pollen placement, because their long stamens could donate pollen efficiently to abundant flowers with long styles in the population and, conversely, their short styles could receive pollen efficiently from abundant flowers with short stamens in the population (Lloyd and Webb, 1992*a*, *b*). In this view, distyly could be considered a solution to the general lack of precise pollen transfer in herkogamous plants (Armbruster *et al.*, 2009), while at the same time limiting the likelihood of geitonogamous self-pollination (Barrett, 2002). There is evidence from experimental manipulations that enantiostyly, particularly the dimorphic form, can reduce levels of geitonogamy (Jesson and Barrett, 2002*b*). However, the prevalence of monomorphic enantiostyly, which can only limit geitonogamy in part, because styles coincide with pollinating stamen positions in half the flowers (Jesson and Barrett, 2003), and the lack of complete reciprocity of sexual organ positions in species of Haemodoraceae with dimorphic enantiostyly (Ornduff and Dulberger, 1978) make the case for enantiostyly as a mechanism of geitonogamy avoidance somewhat less compelling than it is for distyly.

It has been suggested that dimorphic enantiostyly evolved via an intermediate stage of monomorphic enanstiostyly and that style deflection in the monomorphic ancestors preceded the evolution of reciprocal anther positioning (Jesson and Barrett, 2003; Jesson *et al.*, 2003). Selection for deflection of the style in buzz-pollinated plants might be linked with avoidance of physical damage and self-pollination by bees that vibrate the anther cone (Dulberger, 1981), but in other lineages the deflection of the style is more likely to be related to its optimal positioning to contact pollinator surfaces that are pollen rich (Minnaar and Anderson, 2021). Enantiostylous flowers in the Haemodoraceae do not have the same level of reciprocity of the style and pollinating stamens as is found at the flower level in other families, such as the Solanaceae, Fabaceae and Pontederiaceae (Jesson and Barrett, 2003). In *Wachendorfia* (Haemodoraceae), one stamen is deflected to the same side as the style, while two stamens are deflected on the side opposite to the style (Ornduff and Dulberger, 1978). Having one stamen deflected in the same direction as the style is expected to allow for some intra-morph pollen transfer, including geitonogamous self-pollination. This, in turn, could lead to selfed progeny if a self-incompatibility system is absent, as is the case in species such as *Wachendorfia brachyandra* (Jesson and Barrett, 2002a), or even if it is weakly developed (or if early-stage inbreeding depression is evident) as in *Wachendorfia paniculata* L. (Ornduff and Dulberger, 1978). It is particularly difficult to explain why one stamen would be deflected in the same direction as the style if the function of enantiostyly is primarily to reduce geitonogamous self-pollination. One possibility is that this stamen has an important function for intra-morph cross-pollination.

**Fig. 1.**
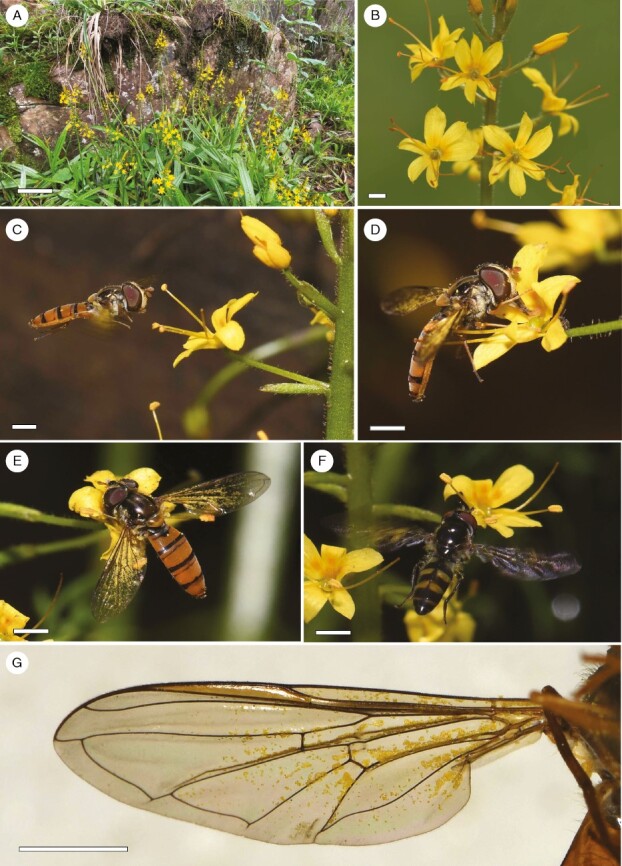
*Barberetta aurea* and syrphid fly pollinators. (A) Plants in their streamside habitat at the Ngele 2 site. (B) Inflorescence of a right-styled morph. (C) The hoverfly *Episyrphus trisectus* approaching flowers. (D) *Episyrphus trisectus* feeding on nectar. (E) Pollen-covered wings of *E. trisectus* in contact with the anthers and stigma. (F) *Melanostoma* sp. (G) Pollen of *B. aurea* on the underside of the wing of *E. trisectus*. Scale bars: 2 mm.

While conducting studies on populations of the rarely seen plant species *Barberetta aurea* Harv (Haemodoraceae), we observed frequent visits by nectar-feeding syrphid flies that carried conspicuous loads of pollen on their wings (Fig. 1). A previous study of the larger-flowered, related species, *W. paniculata*, has shown that pollen deposition occurs on both the wings and the bodies of bees and that pollen transfer by these insects is mostly disassortative among morphs (Minnaar and Anderson, 2021), Here, we consider the possibility that enantiostyly in *B. aurea* has a function to utilize the wings of fly pollinators for pollen transfer. Given that *B. aurea* is sister to the *Wachendorfia* clade (Hopper *et al.*, 2009), establishing its reproductive biology could potentially help to infer the basis for the evolution of enantiostyly in the Haemodoraceae.

The aims of our investigation were as follows: (1) to confirm whether *B. aurea* possesses dimorphic enantiostyly and to document morph ratios in natural populations; (2) to perform controlled pollination experiments to establish whether plants possess self-incompatibility, whether intra- and inter-morph crosses have equal fertility, and whether autonomous self-fertilization is possible; (3) to identify pollinators and establish the mechanisms of pollen transfer; (4) to document floral traits, such as flower colour, scent and nectar properties, that might play a role in attraction of the observed pollinators; and (5) to use colour-labelled pollen analogues to confirm the mechanism of pollen deposition and to estimate the relative likelihood of pollen transfer among and within morphs and how this is affected by the stamen that is deflected in the same direction as the style.

## MATERIALS AND METHODS

### Study species and study sites

**Fig. 2.**
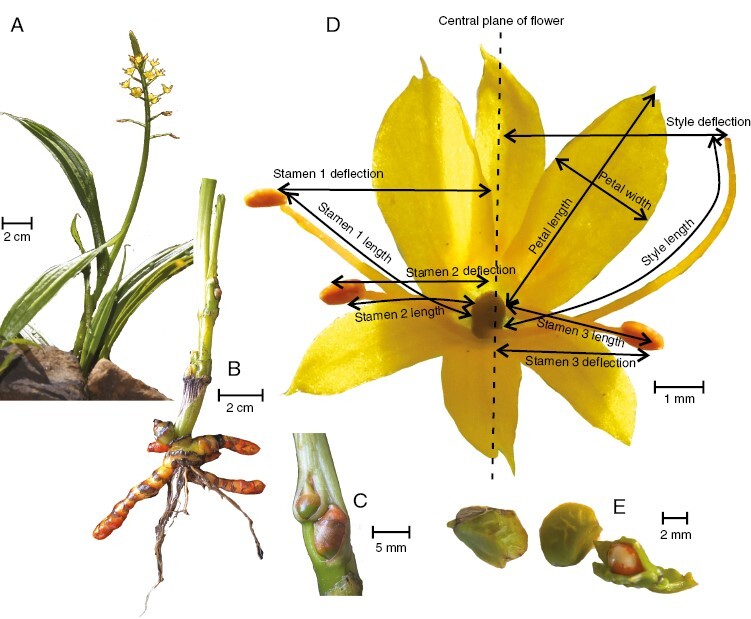
Morphology of *Barberetta aurea*. (A) Flowering plant with plicate leaves. (B) Root system, showing red colour characteristic of Haemodoraceae. (C) Bulbils on the stem. (D) Flower, showing dimensions that were measured. (E) Fruits, including one that is dissected to show the single seed.

*Barberetta aurea* (Figs 1A, B and 2) is a rarely recorded species that occurs in moist habitats, particularly shaded banks of streams, in the Eastern Cape and KwaZulu-Natal provinces of South Africa. Plants are diminutive (usually <30 cm tall) and produce long, slender inflorescences that bear ≤40 flowers with a yellow–orange perianth ~8 mm in diameter and that are unscented to the human nose. The daily mean ± s.e. number of open flowers (display size) per plant is ~5.6 ± 0.25 (*n* *=* 32 plants). Flowers remain open for 2 days and contain a single ovule. The arrangement of floral parts is similar to that in *Wachendorfia*, with the style and one stamen deflected to one side of the flower and two stamens deflected to the other side. Other features shared by *Barberetta* and *Wachendorfia* include plicate leaves (Fig. 2A), yellow–orange flower coloration, red-pigmented rhizomes (Fig. 2B) and overall pollen morphology (Ornduff, 1979; Helme and Linder, 1992). The divergence time for the two clades is estimated at ~15 million years before present (Hopper *et al.*, 2009). *Barberetta aurea* plants develop aerial bulbils on the stems late in the season, which contribute to vegetative reproduction (Fig. 2C). Unlike *Wachendorfia*, which has fruits that dehisce on the plant, the fruits of *B. aurea* (Fig. 2E) are indehiscent and fall from the plant as soon as they reach maturity, suggesting possible dispersal by water along the streamside habitat.

During December–January 2021/2022 and 2022/2023 we studied three *B. aurea* populations (each ~2 km apart) in the upper catchment of the Mtamvuna river above the Merensky timber Weza plantation (30°34ʹS, 29°39ʹE) near the Weza-Ngele forest and one in the catchment of the Karkloof river (29°19ʹS, 30°14ʹE) at Karkloof, which is ~160 km from the Ngele populations. The numbers of flowering plants in each population was ~100 at Ngele 1, 30 at Ngele 2, 5000 at Ngele 3 and 300 at Karkloof. Voucher specimens from both study regions are deposited in the Bews herbarium at the University of KwaZulu-Natal.

### Enantiostyly and morph ratios

To determine whether *B. aurea* has an enantiostylous floral polymorphism and whether this is monomorphic or dimorphic, we recorded style positions (orientation to observer facing the front of the flower) for all open flowers on 76 plants at Karkloof, 117 plants at Ngele 1, 40 plants at Ngele 2, and 299 plants at Ngele 3. These data were also used to calculate morph ratios in these populations.

### Controlled pollination experiments

To establish the compatibility system and pollinator dependence of *B. aurea*, we translocated 60 plants from the streamside Ngele 3 population into pots with wet soil (*n =* 34 plants) or into vases with water (*n =* 27 plants) at the bud stage and kept these plants inside netting enclosures to prevent insects from visiting the flowers. Once flowers on these plants had opened, they were assigned to one of four treatments: (1) unmanipulated, to test for autonomous self-fertilization; (2) manually self-pollinated to test for self-incompatibility or self-compatibility; (3) cross-pollination between plants of the same morph to test for heteromorphic self-incompatibility; and (4) cross-pollination between plants of different morphs to compare with self-pollination and intra-morph cross-pollinations. Each plant received all four treatments in a ‘split plot’ design, and more than one flower per plant was assigned to each treatment in cases where sufficient flowers were available per plant. Because plants reached anthesis at different times, we assigned them to eight different trial groups, each with 7–14 plants, and all plants in a trial group were collected at the same time, kept in the same conditions (either in soil or in water) and were hand-pollinated on the same day. Flowers were hand-pollinated during December 2022 and January 2023, and we scored these flowers for fruit formation 3 weeks after hand-pollinations, when the fruits were swollen and close to full maturity.

### Natural fruit set and stigma pollen loads

We recorded the proportion of flowers that set fruit for both left- and right-styled morphs in the Ngele 3 (*n* = 98 plants) and Karkloof (*n =* 43 plants) populations. We also sampled 55 open flowers from the Ngele population to estimate the percentage of stigmas with pollen deposition and the mean number of pollen grains of *B. aurea* and of other plant species deposited per stigma. Pollen was identified from reference slides.

### Pollinators and pollen loads

We carried out pollinator observations during daylight hours (~09:00–19:00 h) over 11 days with sunny weather covering the full flowering season of the populations at Ngele (6–8 December 2022; 22–23 December 2022; 30–31 December 2022 and 9–10 January 2023) and Karkloof (1 February 2022 and 4 January 2023). Flower visitors were photographed or videoed on flowers and, where possible, captured and stored in individual 5 mL Eppendorf tubes until they could be pinned in the laboratory. From photographs and videos, we were able to determine the species identity, sex and feeding behaviour (nectar vs. pollen feeding) of 92 individual syrphid flies that visited *B. aurea* plants. We used video recordings to determine the number of flowers visited per plant and the duration of visits to individual flowers. Pollen loads on the wings and bodies of flower visitors were counted using a dissecting microscope; the identity of *B. aurea* pollen was confirmed using a compound microscope to compare pollen removed from the bodies of insects (using blocks of Fuchsin gel) with reference slides of pollen removed from the anthers of *B. aurea* flowers (Supplementary Data Fig. S1). Insects captured on flowers of *B. aurea* were measured (i.e. body length and wing length) and identified by taxonomic experts (see Acknowledgements) and deposited in the collection at the University of KwaZulu-Natal.

### Floral traits

To assess the matching of dimensions of flowers and insects, we used digital callipers accurate to 0.1 mm to measure the following floral morphological traits: overall flower width, length of the style and its deflection from the centre of the flower when viewed from the front, length of each anther and their deflection from the centre of the flower when viewed from the front, spatial separation of the anthers and the stigma, petal length and width, the length of anthers, the width of the stigma, and the length and width of the nectar guide (for details, see Fig. 2D).

We recorded floral advertising and reward traits to determine whether these could explain the attraction of specific groups of flower visitors. Spectral reflectance of petals and nectar guides was measured in the 300–700 nm range using an Ocean Optics S2000 spectrometer, as described by Johnson and Anderson (2002). Nectar accumulated in flowers for 24 h was sampled using 5 µL pipettes at both Ngele (*n =* 65 flowers) and Karkloof (*n =* 42 flowers). Owing to the small volumes and high concentration of nectar, we diluted nectar approximately five-fold by adding a known volume of water to the pipettes. The estimated nectar sugar concentration (in grams per 100 g) of the diluted nectar was determined with a refractometer (Bellingham & Stanley model 45-81) and adjusted for the known mass of water added to the pipette.

### Dye particle transfer experiments

To determine whether the floral mechanism of pollen transfer in *B. aurea* favours inter- over intra-morph dispersal, we tracked the transfer of coloured dye particles among flowers in eight experimental trials conducted in small insect flight cages (Bugdorm 2120 width 60 cm × depth 60 cm × height 60 cm) in a laboratory from 1 to 3 January 2023. In each cage we placed four inflorescences from the Ngele 3 population (two left-styled and two right-styled), each with four open flowers, in separate vials filled with water. We applied fluorescent dye powder (Radiant Color, Richmond, CA, USA) to each of the three anthers of flowers of each morph. In the first four trials, the colours assigned to right-styled flowers were blue for anther 1, white for anther 2 and orange–pink for anther 3, and to left-styled flowers, green for anther 1, pink for anther 2 and orange–yellow for anther 3. In four subsequent trials, these colours were reversed among left- and right-styled flowers. Dye particles were applied to anthers with toothpicks until they were fully saturated and additional dye particles did not adhere to the anthers. We placed a single syrphid fly (*Melanostoma* sp. 1, *n =* 7; *Asarkina* aff. *ericotorum*, *n =* 1) in each cage for ~30 min or until the fly had visited all the inflorescences. At the end of each trial, we counted dye particles on the stigmas of each flower under a dissecting microscope, and we pinned each fly and photographed their wings and bodies to assess the distribution of dye particles. Although dye particles are easier to visualize under ultraviolet (UV) light, we used UV light only to identify the presence of dye particles, then counted dye particles under visible illumination because this made it easier to discern colours (white dye particles, for example, tend resemble blue particles under UV light).

### Statistical analyses

Data were analysed using generalized linear models (GLMs) and generalized linear mixed models (GLMMs) with canonical link functions implemented in SPSS v.25 (IBM Corp.). For comparison of morphological traits among plant populations we used Gaussian GLMs. The frequency of morphs and frequency of flowers that set fruit were compared among populations using binomial GLMs. In the analysis of the frequency of flowers that set fruit in the controlled pollination experiments, we used binomial GLMMs, with morph of the recipient plant, source of pollen (self, intra-morph cross and inter-morph cross) and the interaction of recipient morph and pollen donor as fixed effects. Trial and plant nested within trial were treated as random effects to account for the date of pollinations, growth environment and identity of plants used in these experiments. The analysis of pollen loads of flies was based on a negative binomial GLM, with fly species, sex and the interaction of fly species and sex as fixed effects. The analysis of numbers of dye particles on stigmas in the dye-transfer experiments was based on a negative binomial GLMM, with morph of the recipient, morph of the dye donor and the interaction of recipient morph and donor morph as fixed effects. In place of morph of the dye donor, we also tested models using the orientation of donor stamens and donor morph excluding dye particles donated by anther 3 (orientated in the same direction as the style). Random effects were trial and plant nested within trial. To obtain mean counts of dye particles per flower, we used the natural logarithm of the number of flowers per plant as an offset. For graphical presentation of models, we calculated marginal (model-adjusted) means and (asymmetrical) standard errors by back-transformation from the scale used in the link function. Significance testing was based on likelihood ratios (GLMs) or *F*-values (GLMMs). Post-hoc comparison of means was based on the Dunn–Sidak method.

## RESULTS

### Enantiostyly and morph ratios

**Fig. 3.**
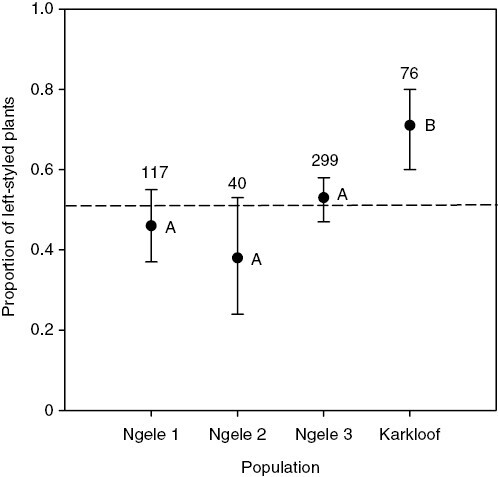
Morph ratios in four populations of *Barberetta aurea*. Values are means and 95 % confidence intervals. The number of plants sampled is indicated above the confidence intervals. Means that share letters are not significantly different.

Across the four populations, we found that style positions were fixed to either the left or the right in 99.4 % of the 534 plants surveyed, indicating a system of dimorphic enantiostyly. Although morph frequencies varied significantly among populations (χ^2^ = 16.4, *P* < 0.001), they did not differ significantly from 50:50 in the three Ngele populations and were significantly left-biased (71 %) only at Karkloof (Fig. 3).

### Controlled pollination experiments

**Fig. 4.**
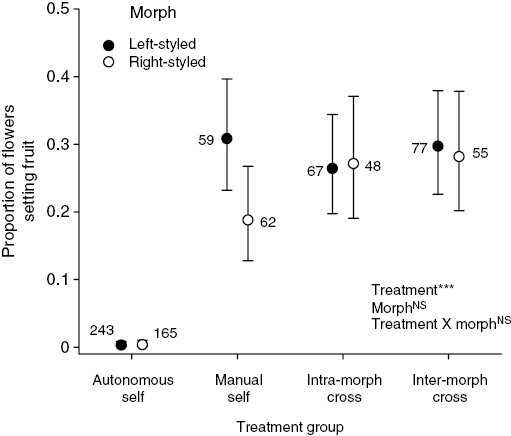
Results of controlled hand-pollinations to determine the type of genetic compatibility system and level of autonomous self-fertilization in *B. aurea*. Values are means ± s.e. Numbers indicate the sample size of flowers in each treatment group. ***P < 0.0001, NS = non-significant.

In the controlled hand-pollination experiments, we found significant effects of treatment (*F* = 14.0, *P* < 0.001), but not of morph (*F* = 0.60, *P* = 0.80) or morph × treatment (*F* = 0.45, *P* = 0.71). There were no significant differences in fruit set among flowers of either morph according to whether they were manually self-pollinated, intra-morph cross-pollinated or inter-morph cross-pollinated (Fig. 4), indicating that the plants of *B. aurea* we investigated were completely self-compatible and lacked heteromorphic incompatibility. Flowers that were not manipulated and were excluded from pollinators failed to set fruits, indicating that *B. aurea* is incapable of autonomous self-pollination and is thus fully dependent on pollinator visits for seed production.

### Stigma pollen loads and natural fruit set

The mean ± s.e. number of *B. aurea* pollen grains deposited per stigma at Karkloof was 1.94 ± 0.37 (range 0–10, *n* = 55 stigmas) and did not differ among morphs (χ^2^ = 1.19, *P* = 0.27). The mean length of pollen grains on stigmas was 43.8 ± 1.42 µm and did not differ among morphs (χ^2^ = 0.32, *P* = 0.56). Pollen grains observed on stigmas closely matched the surface sculpturing and length (39.5 ± 0.45 µm) of reference pollen grains of *B. aurea* (Supplementary Data Fig. S1), and we did not observe any heterospecific pollen grains on stigmas. The diameter of the stigma of *B. aurea* is 121.9 ± 2.94 µm, which is three times greater than the length of the pollen grains. A maximum of ten pollen grains were observed to fit on a stigma. Flowers produce a single ovule. The mean ± s.e. percentage of flowers that set fruit naturally was almost ten-fold greater at Ngele site 3 than at Karkloof (60.3 ± 15.6 vs. 7.2 ± 0.8, χ^2^ = 613.8, *P *< 0.0001), but did not differ according to morph (χ^2^ = 0.31, *P* = 0.57) or the interaction of site and morph (χ^2^ = 0.54, *P* = 0.46).

### Pollinators and their pollen loads

**Fig. 5.**
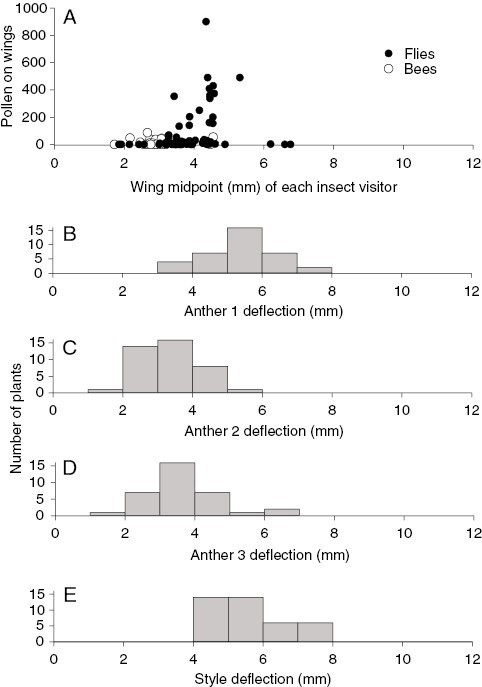
Number of *Barberetta aurea* pollen grains on insects with different wing lengths in relationship to the deflection of stamens and styles of *B. aurea*.

Over a period of ~88 h, we observed ~400 insect visitors on flowers of *B. aurea*, of which ~90 % were syrphid flies representing at least eight species (Table 1). The most abundant syrphid species among all filmed and captured individuals were *Melanostoma* sp. 1 (*n* = 101), *Episyrphus trisectus* (*n* = 25), *Allograpta fuscotibialis* (*n* = 10) and *Betasyrphus adlagiatus* (*n* = 5). Syrphid flies contacted the anthers and stigmas with their wings while hovering in front of flowers before settling to feed (Fig. 1C–F; Supplementary Data Video S1). For syrphid fly individuals (*n* = 92) that were photographed or filmed to record details of their foraging behaviour, nectar feeding was observed for the great majority (79 %) of individuals, with the remainder (21 %) feeding on pollen. The percentage of individuals feeding on pollen did not differ among syrphid fly species (χ^2^ = 1.38, *P *= 0.50). We recorded 66 females and 91 males among the syrphid individuals that were filmed or captured on flowers. The percentage of syrphid flies that fed on pollen was significantly higher for females (28 %) than it was for males (9 %; χ^2^ = 4.71, *P *= 0.03). The mean ± s.e. number of flowers visited per plant by syrphids was 1.63 ± 0.15, and the duration of their visits to individual flowers was 5.22 ± 0.45 s. Syrphid flies dominated the assemblage of 97 insect individuals that we captured on flowers of *B. aurea* (Table 1). The wings of syrphids had conspicuous pollen loads (Fig. 1C–F), and examination of captured specimens showed that this pollen matched reference collections of *B. aurea* pollen. Pollen loads on wings (Table 1) differed significantly among fly genera (χ^2^ = 18.26, *P *< 0.001), but not according to fly sex (χ^2^ = 1.22, *P *= 0.26) or the interaction of genus and sex (χ^2^ = 2.52, *P *= 0.28). The largest pollen loads were found on syrphid flies with wings 8–10 mm in length (Table 1; Fig. 5A). Honeybees were absent from the large Ngele 3 site, but were present in low numbers (~20 individuals observed) at the Karkloof site and carried small pollen loads (Table 1). Small pollen-collecting bees were observed at Ngele and Karkloof, but these also carried very small amounts of pollen (Table 1).

**Table 1.** Dimensions and pollen loads of insects captured on flowers of *Barberetta aurea*. Values are means ± s.e. Site: N = Ngele, K = Karkloof. The largest pollen loads on wings are indicated in bold.

Taxon	Site	*n*	Body length (mm)	Wing length (mm)	*B. aurea* pollen on wings	*B. aurea* pollen on body
Empididae						
*Empis*	N	1	3.2 ± 0.00	3.4 ± 0.00	0.0 ± 0.0	0.0 ± 0.0
Syrphidae						
*Allograpta fuscotibialis*	N	8	8.8 ± 0.42	8.3 ± 0.36	**102.7 ± 36.5**	29.6 ± 11.3
*Asarkina aff ericetorum*	N	3	13.4 ± 0.70	13.8 ± 0.59	0 ± 0.00	18.0 ± 13.1
*Betasyrphus adligatus*	N	3	9.5 ± 0.70	9.3 ± 0.59	**303.0 ± 175.2**	68.3 ± 39.7
*Episyrphus trisectus*	N, K	5	8.9 ± 0.54	8.7 ± 0.46	**410.6 ± 183.8**	19.2 ± 8.8
*Melanostoma* sp. 1	N, K	46	7.5 ± 0.17	7.5 ± 0.15	41.2 ± 6.6	15.8 ± 2.6
*Melanostoma* sp. 2	R	3	9.1 ± 1.21	8.8 ± 1.03	**168.3 ± 97.4**	1.5 ± 1.6
*Paragus longiventris*	R	1	4.8 ± 0.00	3.7 ± 0.00	0.0 ± 0.00	4.0 ± 0.00
*Paragus tibialis*	N, K	2	5.0 ± 0.85	4.5 ± 0.73	0.0 ± 0.0	0.0 ± 0.0
Tabanidae						
*Philoliche* sp. 1	N	1	8.6 ± 0.00	12.4 ± 0.00	0.0 ± 0.0	0.0 ± 0.0
Tachinidae						
*Tachinidae* sp. 1	N, K	2	5.1 ± 1.21	4.3 ± 1.03	0.0 ± 0.0	0.0 ± 0.0
Halicitidae						
*Patellapis* sp. 1	N	5	5.8 ± 0.54	5.3 ± 0.46	20.0 ± 9.1	164.0 ± 73.5
*Patellapis* sp. 2	N	6	6.6 ± 0.45	5.7 ± 0.42	12.0 ± 5.1	47.2 ± 19.4
Apidae						
Honeybee	K	2	11.7 ± 0.00	9.1 ± 0.00	54.0 ± 54.4	140.0 ± 140.4
*Braunsapis* sp.	K	11	7.4 ± 0.36	8.6 ± 0.73	16.4 ± 4.8	36.9 ± 11.2

### Floral traits

**Fig. 6.**
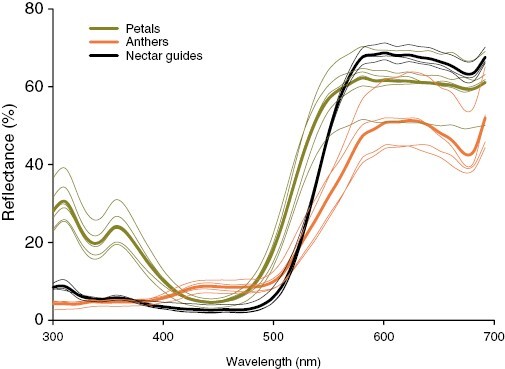
Spectral reflectance of parts of flowers of *Barberetta aura*. Bold lines indicate mean values.

Apart from the directions of style and stamen deflection, we observed no consistent differences in morphology between the two morphs (Table 2). There were, however, some differences in morphology among sites, with plants at Ngele having broader dorsal petals, larger nectar guides and slightly shorter stamens and styles that are not deflected as far to the left of right of the central axis of the flower, in comparison to those at Karkloof. The deflection of the style and stamens correspond closely to the sites of pollen deposition on the wings of syrphid flies (Fig. 5). Pronounced stigma–anther separation, and the tendency during flower wilting for the style to remain rigid while the stamens shrivel, explains the lack of autonomous self-fertilization in this species. Although petals and nectar guides of *B. aurea* do not contrast strongly in human-visible wavelengths, the petals are strongly UV-reflecting, whereas the nectar guides are UV-absorbing (Fig. 6). The overall spectral reflectance of the nectar guides is similar to that of the anthers (Fig. 6). Flowers of *B. aurea* contained on average ~0.5 µL of nectar with a relatively high sugar concentration (in grams per 100 g) of 40–50 % (for details, see Table 2).

**Table 2.** Morphological traits and nectar properties of *Barberetta aurea*. Values are means ± s.e. All units are millimetres unless otherwise stated. See Materials and Methods section for sample sizes. *P < 0.05, **P < 0.01; NS = not significant.

Trait	Ngele		Karkloof		Fixed effects		
	Left-styled	Right-styled	Left-styled	Right-styled	Site	Morph	Site × morph
Flower width	7.4 ± 0.38	7.0 ± 0.38	8.1 ± 0.33	7.3 ± 0.34	NS	NS	NS
Petal length	4.6 ± 0.17	4.9 ± 0.19	5.1 ± 0.22	5.1 ± 0.22	NS	NS	NS
Petal width	2.7 ± 0.09	2.7 ± 0.10	1.8 ± 0.12	1.9 ± 0.12	**	NS	NS
Nectar guide	1.3 ± 0.07	1.4 ± 0.08	1.1 ± 0.09	1.1 ± 0.09	*	NS	NS
Style length	6.5 ± 0.20	6.4 ± 0.22	8.0 ± 0.24	7.7 ± 0.22	**	NS	NS
Stamen 1 length	6.9 ± 0.24	7.1 ± 0.23	7.8 ± 0.28	7.8 ± 0.27	**	NS	NS
Stamen 2 length	6.1 ± 0.21	6.3 ± 0.23	7.8 ± 0.29	6.6 ± 0.27	**	NS	NS
Stamen 3 length	6.4 ± 0.19	6.3 ± 0.21	7.8 ± 0.26	6.6 ± 0.24	**	**	*
Style diversion	4.1 ± 0.21	5.4 ± 0.23	5.7 ± 0.27	5.9 ± 0.27	**	NS	NS
Stamen 1 deflection	4.8 ± 0.21	5.5 ± 0.23	5.7 ± 0.27	5.9 ± 0.27	**	NS	NS
Stamen 2 deflection	2.9 ± 0.18	3.2 ± 0.20	4.4 ± 0.24	3.6 ± 0.24	**	NS	*
Stamen 3 deflection	3.6 ± 0.19	3.2 ± 0.21	4.6 ± 0.24	3.4 ± 0.24	**	**	NS
Stigma–anther 3	3.4 ± 0.36	3.3 ± 0.36	4.6 ± 0.31	3.2 ± 0.31	NS	*	NS
Nectar volume (µL)	0.50 ± 0.03	0.52 ± 0.03	0.40 ± 0.05	0.42 ± 0.04	*	NS	NS
Nectar concentration (%)	50.3 ± 3.34	52.9 ± 3.79	40.1 ± 4.55	42.7 ± 4.34	*	NS	NS

### Dye particle transfer experiments

**Fig. 7.**
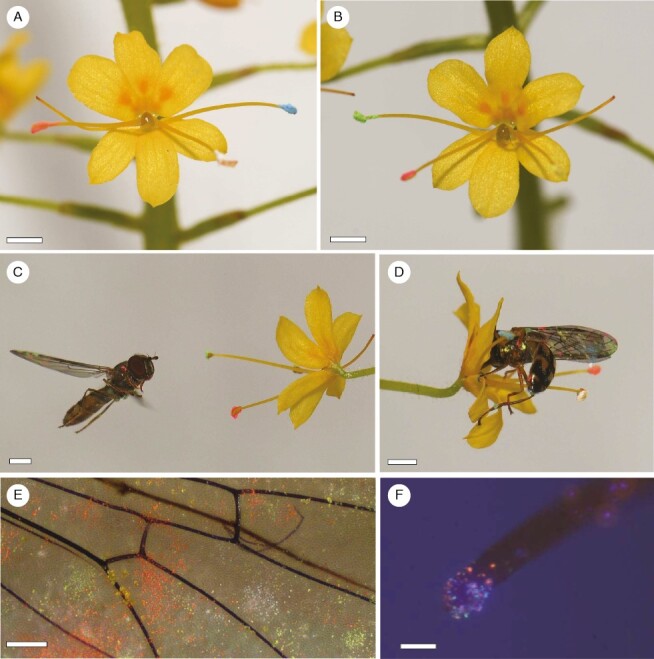
Transfer of dye particles on the wings of syrphid flies visiting flowers of *Barberetta aurea*. (A) Dye particles applied to anthers of a left-styled morph. (B) Dye particles applied to anthers of a right-styled morph. (C) *Melanostoma* sp. with dye particles on wings approaching a flower. (D) *Melanostoma* sp. 1. feeding on nectar of flower with dye particles. (E) Wing of *Melanostoma* sp. 1 at the end of a trial, showing large numbers of attached dye particles and pollen grains. (F) Dye particles on a stigma. Scale bars: 2 mm in A–D; 500 µm in E; 200 µm in F.

**Fig. 8.**
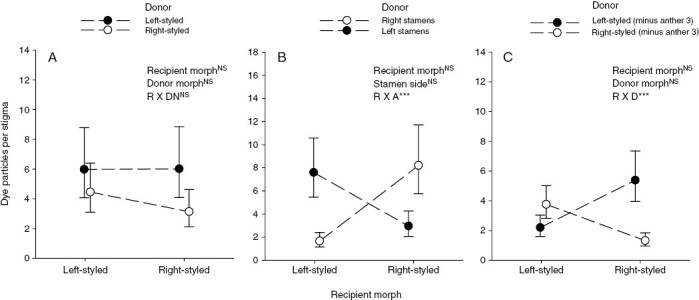
Patterns of dye particle dispersal among flowers of *Barberetta aurea*. (A) Dispersal according to morph. (B) Dispersal according to stamen orientation. (C) Dispersal according to morph, excluding anther 3, which is oriented in the same direction as the style. ***P < 0.001, NS = not significant.

Dye particles were conspicuous on the wings of the syrphid flies that were used in the cage experiments (Fig. 7) and were transferred to 67 % of the stigmas of flowers used in these experiments. There was no evidence for an overall pattern of disassortative dye transfer between the two morphs (morph by donor interaction: *F* = 0.004, *P *= 0.95; Fig. 8A). However, dye received by left-styled morphs was more likely to originate from left-facing stamens, and vice versa for right-styled morphs (morph by donor interaction: *F* = 13.4, *P* < 0.001; Fig. 8B). We detected a significant pattern of disassortative dye transfer when dye from anther 3, which is orientated to the same side as the style, was excluded from the analysis (morph by donor interaction: *F* = 10.4, *P* = 0.002; Fig. 8C).

## DISCUSSION

The results of our study revealed that flowers of *B. aurea* are pollinated primarily by syrphid flies, which were abundant in the shaded streamside habitats occupied by populations of this plant species. Large loads of pollen were evident on the wings of captured and photographed flies, and dye particles applied to anthers were transferred to the wings of syrphid flies (and subsequently to stigmas) in cage experiments. The deflected stamens and style contact the wings of flies as they approach and feed on flowers, and pollen loads on stigmas were entirely conspecific. Although some visits by small solitary bees and honeybees were observed, these insects were far less frequent visitors than flies, tended to focus on pollen collection from the anthers, and carried far fewer pollen grains on their wings than did syrphid flies (Table 1), which dominated the visitor assemblages in all the study populations. It is notable that the highest level of fruit set (60 % of flowers) was recorded for the Ngele 3 site, where honeybees were absent as visitors. These observations confirm that the mechanism of pollen transfer between flowers of *B. aurea* primarily involves syrphid fly pollination, and to our knowledge, this pollination system has not been reported previously in an enantiostylous species.

Apart from the matching of the floral morphology of *B. aurea* to the wings of syrphid flies, several features of the flowers are consistent with traits that have been shown to play a functional role in other fly pollination systems. These include the presentation of concentrated (40–50 %) nectar in a thin film (Johnson *et al.*, 2020). Syrphids are known to probe instinctively at nectar guides with spectral properties similar to pollen (Lunau *et al.*, 2018), and this was evident in their behaviour on flowers of *B. aurea*, which have UV-absorbing nectar guides that contrast with the UV-reflecting tepals (Figs 1C and 6). Syrphid flies are commonly reported as components of pollinator assemblages of species with a generalized pollination system and are generally considered to have low effectiveness as pollinators because of their relatively hairless bodies (Sahli and Conner, 2007; Bischoff *et al.*, 2013). Syrphids frequently feed on pollen (Gilbert, 1981), which might further reduce their effectiveness as pollinators, but this behaviour occurred in a relative small proportion (21 %) of visits to flowers of *B. aurea*, perhaps on account of the readily accessible nectar associated with a conspicuous nectar guide and the long stamen filaments, which make it difficult for syrphids to probe at the anthers while perched on the flower. It is unusual for a plant species to exhibit dependence on, or apparent specialization for, pollination by syrphids. Apart from some orchids pollinated by syrphids deceived by floral mimicry of their brood sites (Johnson and Schiestl, 2016), there are very few other plant species for which syrphids have been reported to be the primary pollinators (Lindsey, 1984; McGuire and Armbruster, 1991).

Adaptations of plants for transfer of pollen on insect wings is increasingly being recognized as being an important factor in the evolution of complex floral morphology (Butler and Johnson, 2020; Daniels *et al.*, 2020). Wing pollination has now been recorded in at least eight plant families and is strongly associated with highly exserted styles and stamens, often in a brush arrangement (Butler and Johnson, 2020). It has been reported previously for flowers pollinated by butterflies (Cruden and Hermann-Parker, 1979; Kiepiel and Johnson, 2014; Epps *et al.*, 2015; Butler and Johnson, 2020; Daniels *et al.*, 2020) and bees (Holmqvist *et al.*, 2005; Minnaar and Anderson, 2021), but the present study is, to our knowledge, the first known case involving flies. The traits associated with wing pollination systems are not fully elucidated, but one common feature appears to be pollen grains that are very sticky. By brushing a coverslip over anthers of *B. aurea*, we found that pollen grains adhered strongly to the glass and could not be removed by vigorous shaking of the glass; a similar process of adhesion probably occurs between the pollen and the beating wings of syrphid flies. The stigma of *B. aurea* is covered with a sticky exudate, and the forces of adhesion between the stigma and the pollen must be even greater than those between the pollen and the wings of the flies.

The mostly equal morph ratios in populations of *B. aurea* are likely to be maintained by negative frequency-dependent selection owing to disassortative mating among morphs. Although we were not able to detect disassortative transfer of dye particles in our experiments (Fig. 8A), it is likely to occur, because two of the three stamens are positioned reciprocally to the style. However, we did find, as expected, that dye particle transfer occurred mostly between reciprocally positioned floral organs (Fig. 8B, C). This confirmed that intra-morph pollination was attributable mainly to the contribution of the single stamen that is orientated in the same direction as the style.

Our dye particle experiments were mostly conducted using *Melanostoma* sp. 1 flies because they were the most abundant visitor species, but these flies carried relatively small pollen loads compared with some of the other syrphid visitor species (Table 1), perhaps because they fold their wings after landing on flowers (Fig. 7D). Syrphid species such as *E. trisectus* and *Betasyrphus adligatus*, which carried larger pollen loads (Fig. 1C–G; Table 1; Supplementary Data Video S1), keep their wings open when feeding on nectar in flowers and are thus likely to be more effective pollinators. Another caveat to our dye-transfer experiments is that we were unable to distinguish between self and cross intra-morph pollination, because the flies circulated continuously among the four inflorescences in each cage. The dye particles used in our experiment are much smaller than pollen grains and do not mimic pollen carryover properties accurately (Thomson *et al.*, 1986). We used dye particles because, unlike alternative pollen labelling methods, such as quantum dots, dye particles could be obtained in six unique colours, which was important for tracking pollen from individual anthers. Dye particles were considered adequate for confirming the site of pollen deposition on wings and for comparing the relative amount of intra-morph vs. intermorph dye transfer, but not as a proxy for the total amount of pollen that flies would transfer. Because of the various caveats to our experimental design, it would be valuable to repeat these experiments using more effective pollinators, such as *E. trisectus*, which carried larger pollen loads (Fig. 1; Table 1), using direct colour labelling of pollen, such as quantum dots (Minnaar and Anderson, 2019), in order to test more accurately for disassortative pollen transfer in this species, and also by using a design that allows for self vs. cross intra-morph pollination to be distinguished.

It would also be of considerable interest to quantify the mating system of *B. aurea* using genetic markers. This would be important for at least two reasons. First, our controlled pollination experiments demonstrated that plants are strongly self-compatible and thus we might expect some selfing to occur via geitonogamous self-pollination, given that plants display several open flowers during most of the flowering period and have one stamen deflected to the same side as the style. Second, quantifying levels of disassortative mating using genetic markers would also be valuable to confirm results from our dye experiments. Using allozyme markers, Jesson and Barrett (2002*a*) found that rates of outcrossing in *W. paniculata* were generally high (>80 %), despite the potential contribution of the stamens orientated in the same direction as the style. Minaar and Anderson (2021) found that pollen transfer among *W. paniculata* flowers was mostly disassortative and that stamen 3 played only a small role in overall pollen transfer, but their results could be specific to the morphometrics of the particular insect species used in their experiments. Our results indicate that stamen 3 of *B. aurea* could play a significant, even disproportionate, role in overall pollen transfer and could thus be involved in geitonogamy. However, the probability of newly deposited self-pollen grain being captured by the plant’s own stigma through geitonogamy might be very low, on account of the large surface on which pollen is deposited on the wings relative to the dimensions of the pin-like stigma. This might be an example where lack of precision (*sensu*Armbruster *et al.*, 2004) in the sites of pollen deposition and pick-up could promote outcrossing via extensive pollen carryover. The prediction of extensive pollen carryover in *B. aurea* could be tested easily through experimentation, by using deposition of pollen from a donor over a series of emasculated flowers (Waser and Price, 1984; Morris *et al.*, 1994) or by using quantum dot pollen labelling of donor plants in natural populations. We predict that stamen 3 poses little risk for self-fertilization and pollen discounting (loss of siring opportunities owing to self-pollination) for this plant and that its main function is to contribute to valuable intra-morph cross-pollination.

Some have argued that lack of complete reciprocity in male and female organ positioning (as is the case for *B. aurea*) can represent a form of reproductive assurance through pollinator-mediated geitonogamy. Mora-Carrera *et al*. (2019), for example, argued that monomorphic enantiostyly might be maintained under pollen limitation because it would increase the probability of geitonogamy. We do not agree with their argument, because reproductive assurance through selfing would be achieved more efficiently through a system of delayed self-fertilization. In the case of *B. aurea*, it seems more likely that incomplete reciprocity in organ positioning is driven by the overall advantages of exploiting both wings of a visiting insect and thus the ability to export pollen to both morphs in the population and thereby contribute to overall levels of cross-pollination. From a female perspective, it is clear that styles are deflected to contact pollen-rich parts of animal visitors. There are other wing-pollinated plant species, such as *Hesperantha coccinea* (Iridaceae), that have highly ranched styles with lobes deflected to both the left and right of the flower, such that they can receive pollen from both wings of visiting butterflies (Johnson and Bond, 1994). Enantiostyly in the Haemodoraceae might thus be considered an alternative solution to highly branched styles as a means of receiving pollen effectively from the wings of insect visitors.

## SUPPLEMENTARY DATA

Supplementary data are available at *Annals of Botany* online and consist of the following.

Fig. S1: pollen grains of *Barberetta aurea* in relationship to the very small stigma of this species. Video S1: insect visits to flowers of *Barberetta aurea*.

mcad118_suppl_Supplementary_Figure

mcad118_suppl_Supplementary_Video

## References

[CIT0001] Armbruster S , Pélabon C, Hansen T, Mulder C. 2004. Floral integration, modularity, and accuracy: distinguishing complex adaptations from genetic constraints. In: Phenotypic integration: studying the ecology and evolution of complex phenotypes. Oxford University Press, 23–49.

[CIT0002] Armbruster WS , Hansen TF, Pelabon C, Perez-Barrales R, Maad J. 2009. The adaptive accuracy of flowers: measurement and microevolutionary patterns. Annals of Botany 103: 1529–1545. doi:10.1093/aob/mcp095. 19429671 PMC2701747

[CIT0003] Barrett SCH. 1992. Evolution and function of heterostyly. New York: Springer-Verlag.

[CIT0004] Barrett SCH. 2002. The evolution of plant sexual diversity. Nature Reviews Genetics 3: 274–284. doi:10.1038/nrg776. 11967552

[CIT0005] Barrett SCH. 2010. Darwin’s legacy: the forms, function and sexual diversity of flowers. Philosophical Transactions of the Royal Society B: Biological Sciences 365: 351–368. doi:10.1098/rstb.2009.0212. PMC2838255 20047864

[CIT0006] Barrett SCH , Cruzan MB. 1994. Incompatibility in heterostylous plants. In: Williams EG, Clarke AE, Knox RB, eds. Genetic control of self-incompatibility and reproductive development in flowering plants. Dordrecht: Kluwer Academic, 189–219.

[CIT0007] Bischoff M , Campbell DR, Lord JM, Robertson AW. 2013. The relative importance of solitary bees and syrphid flies as pollinators of two outcrossing plant species in the New Zealand alpine. Austral Ecology 38: 169–176. doi:10.1111/j.1442-9993.2012.02389.x.

[CIT0008] Butler HC , Johnson SD. 2020. Butterfly-wing pollination in *Scadoxus* and other South African Amaryllidaceae. Botanical Journal of the Linnean Society 193: 363–374. doi:10.1093/botlinnean/boaa016.

[CIT0009] Cruden RW , Hermann-Parker SM. 1979. Butterfly pollination of *Caesalpinia pulcherrima*, with observations on a psychophilous syndrome. Journal of Ecology 67: 155–168. doi:10.2307/2259342.

[CIT0010] Daniels RJ , Johnson SD, Peter CI. 2020. Flower orientation in *Gloriosa superba* (Colchicaceae) promotes cross-pollination via butterfly wings. Annals of Botany 125: 1137–1149. doi:10.1093/aob/mcaa048. 32188969 PMC7262471

[CIT0011] Darwin C. 1877. The different forms of flowers on plants of the same species. London: Murray.

[CIT0012] Dulberger R. 1981. The floral biology of *Cassia didymobotrya* and *C. auriculata* (Caesalpiniaceae). American Journal of Botany 68: 1350–1360. doi:10.1002/j.1537-2197.1981.tb07846.x.

[CIT0013] Dulberger R , Ornduff R. 1980. Floral morphology and reproductive biology in four species of *Cyanella* (Tecophilaeaceae). New Phytologist 86: 45–56. doi:10.1111/j.1469-8137.1980.tb00778.x.

[CIT0014] Epps MJ , Allison SE, Wolfe LM. 2015. Reproduction in flame azalea (*Rhododendron calendulaceum*, Ericaceae): a rare case of insect wing pollination. The American Naturalist 186: 294–301. doi:10.1086/682006. 26655157

[CIT0015] Ganders FR. 1979. The biology of heterostyly. New Zealand Journal of Botany 17: 607–635. doi:10.1080/0028825x.1979.10432574.

[CIT0016] Gilbert FS. 1981. Foraging ecology of hoverflies: morphology of the mouthparts in relation to feeding on nectar and pollen in some common urban species. Ecological Entomology 6: 245–262. doi:10.1111/j.1365-2311.1981.tb00612.x.

[CIT0017] Helme NE , Linder HP. 1992. Morphology, evolution and taxonomy of *Wachendorfia* Haemodoraceae. Bothalia 22: 59–57. doi:10.4102/abc.v22i1.826.

[CIT0018] Holmqvist J-H , Manktelow M, Daniel TF. 2005. Wing pollination by bees in *Mexacanthus* (Acanthaceae)? Acta Botanica Mexicana 71: 11–17.

[CIT0019] Hopper SD , Smith RJ, Fay MF, Manning JC, Chase MW. 2009. Molecular phylogenetics of Haemodoraceae in the Greater Cape and Southwest Australian floristic regions. Molecular Phylogenetics and Evolution 51: 19–30. doi:10.1016/j.ympev.2008.11.015. 19063982

[CIT0020] Jesson LK , Barrett SCH. 2002a. Enantiostyly in *Wachendorfia* (Haemodoraceae): the influence of reproductive systems on the maintenance of the polymorphism. American Journal of Botany 89: 253–262. doi:10.3732/ajb.89.2.253. 21669734

[CIT0021] Jesson LK , Barrett SCH. 2002b. Enantiostyly: solving the puzzle of mirror-image flowers. Nature 417: 707–707. doi:10.1038/417707a. 12066175

[CIT0022] Jesson LK , Barrett SCH. 2002c. The genetics of mirror-image flowers. Proceedings of the Royal Society B: Biological Sciences 269: 1835–1839. doi:10.1098/rspb.2002.2068. PMC1691103 12350272

[CIT0023] Jesson LK , Barrett SCH. 2003. The comparative biology of mirror-image flowers. International Journal of Plant Sciences 164: S237–S249. doi:10.1086/378537.

[CIT0024] Jesson LK , Barrett SCH. 2005. Experimental tests of the function of mirror-image flowers. Biological Journal of the Linnean Society 85: 167–179. doi:10.1111/j.1095-8312.2005.00480.x.

[CIT0025] Jesson LK , Barrett SCH, Day T. 2003. A theoretical investigation of the evolution and maintenance of mirror-image flowers. The American Naturalist 161: 916–930. doi:10.1086/375176. 12858276

[CIT0026] Johnson SD , Andersson S. 2002. A simple field method for manipulating ultraviolet reflectance of flowers. Canadian Journal of Botany-Revue Canadienne De Botanique 80: 1325–1328. doi:10.1139/b02-116.

[CIT0027] Johnson SD , Bond WJ. 1994. Red flowers and butterfly pollination in the fynbos of South Africa. In: Arianoutsou M, Groves RH eds. Plant–animal interactions in Mediterranean-type ecosystems. Dordrecht: Kluwer Academic 137–148.

[CIT0028] Johnson SD , Schiestl FP. 2016. Floral mimicry. Oxford: Oxford University Press.

[CIT0029] Johnson SD , Sivechurran J, Doarsamy S, Shuttleworth A. 2020. Dung mimicry: the function of volatile emissions and corolla patterning in fly-pollinated *Wurmbea* flowers. New Phytologist 228: 1662–1673. doi:10.1111/nph.16791. 33460187

[CIT0030] Kiepiel I , Johnson SD. 2014. Shift from bird to butterfly pollination in *Clivia* (Amaryllidaceae). American Journal of Botany 101: 190–200. doi:10.3732/ajb.1300363. 24414430

[CIT0031] Lindsey AH. 1984. Reproductive biology of Apiaceae. I. Floral visitors to *Thaspium* and *Zizia* and their importance to pollination. American Journal of Botany 71: 375–387. doi:10.1002/j.1537-2197.1984.tb12524.x.

[CIT0032] Lloyd DG , Webb CJ. 1992a. The evolution of heterostyly. In: Barrett SCH, ed. Evolution and function of heterostyly. Berlin: Springer-Verlag, 151–178.

[CIT0033] Lloyd DG , Webb CJ. 1992b. The selection of heterostyly. In: Barrett SCH, ed. Evolution and function of heterostyly. Berlin: Springer-Verlag, 179–207.

[CIT0034] Lunau K , An L, Donda M, Hohmann M, Sermon L, Stegmanns V. 2018. Limitations of learning in the proboscis reflex of the flower visiting syrphid fly *Eristalis tenax*. PLoS One 13: e0194167. doi:10.1371/journal.pone.0194167. 29558491 PMC5860702

[CIT0035] McGuire AD , Armbruster WS. 1991. An experimental test for reproductive interactions between two sequentially blooming *Saxifraga* species (Saxifragaceae). American Journal of Botany 78: 214–219.

[CIT0036] Minnaar C , Anderson B. 2019. Using quantum dots as pollen labels to track the fates of individual pollen grains. Methods in Ecology and Evolution 10: 604–614. doi:10.1111/2041-210x.13155.

[CIT0037] Minnaar C , Anderson B. 2021. A combination of pollen mosaics on pollinators and floral handedness facilitates the increase of outcross pollen movement. Current Biology 31: 3180–3184.e3. doi:10.1016/j.cub.2021.04.074. 34043951

[CIT0038] Mora-Carrera E , Castañeda-Zárate M, Fornoni J, Boege K, Domínguez CA. 2019. On the adaptive value of monomorphic versus dimorphic enantiostyly in *Solanum rostratum*. Annals of Botany 123: 205–212. doi:10.1093/aob/mcy162. 30184097 PMC6344091

[CIT0039] Morris WF , Price MV, Waser NM, Thomson JD, Thomson B, Stratton DA. 1994. Systematic increase in pollen carryover and its consequences for geitonogamy in plant populations. Oikos 71: 431–440. doi:10.2307/3545831.

[CIT0040] Ornduff R. 1979. Chromosome numbers and relationships of certain African and American genera of Haemodoraceae. Annals of the Missouri Botanical Garden 66: 577–580. doi:10.2307/2398851.

[CIT0041] Ornduff R , Dulberger R. 1978. Floral enantiomorphy and the reproductive system of *Wachendorfia paniculata* (Haemodoraceae). New Phytologist 80: 427–434. doi:10.1111/j.1469-8137.1978.tb01577.x.

[CIT0042] Richards AJ. 1997. Plant breeding systems, 2nd edn. London: Chapman & Hall.

[CIT0043] Sahli HF , Conner JK. 2007. Visitation, effectiveness, and efficiency of 15 genera of visitors to wild radish, *Raphanus raphanismum* (Brassicaceae). American Journal of Botany 94: 203–209. doi:10.3732/ajb.94.2.203. 21642222

[CIT0044] Thomson JD , Price MV, Waser NM, Stratton DA. 1986. Comparative studies of pollen and fluorescent dye transport by bumble bees visiting *Erythronium grandiflorum*. Oecologia 69: 561–566. doi:10.1007/BF00410363. 28311616

[CIT0045] Waser NM , Price MV. 1984. Experimental studies of pollen carryover: effects of floral variability in *Ipomopsis aggregata*. Oecologia 62: 262–268. doi:10.1007/BF00379024. 28310724

