# Supplementary material for: The enantiostylous floral polymorphism of Barberetta aurea (Haemodoraceae) facilitates wing pollination by syrphid flies
Source: Ann Bot. 2023 Aug 26;132(6):1107–18. doi: 10.1093/aob/mcad118 (PMC10809052; doi:10.1093/aob/mcad118)
Supplement: mcad118_suppl_Supplementary_Figure [file mcad118_suppl_supplementary_figure.docx]

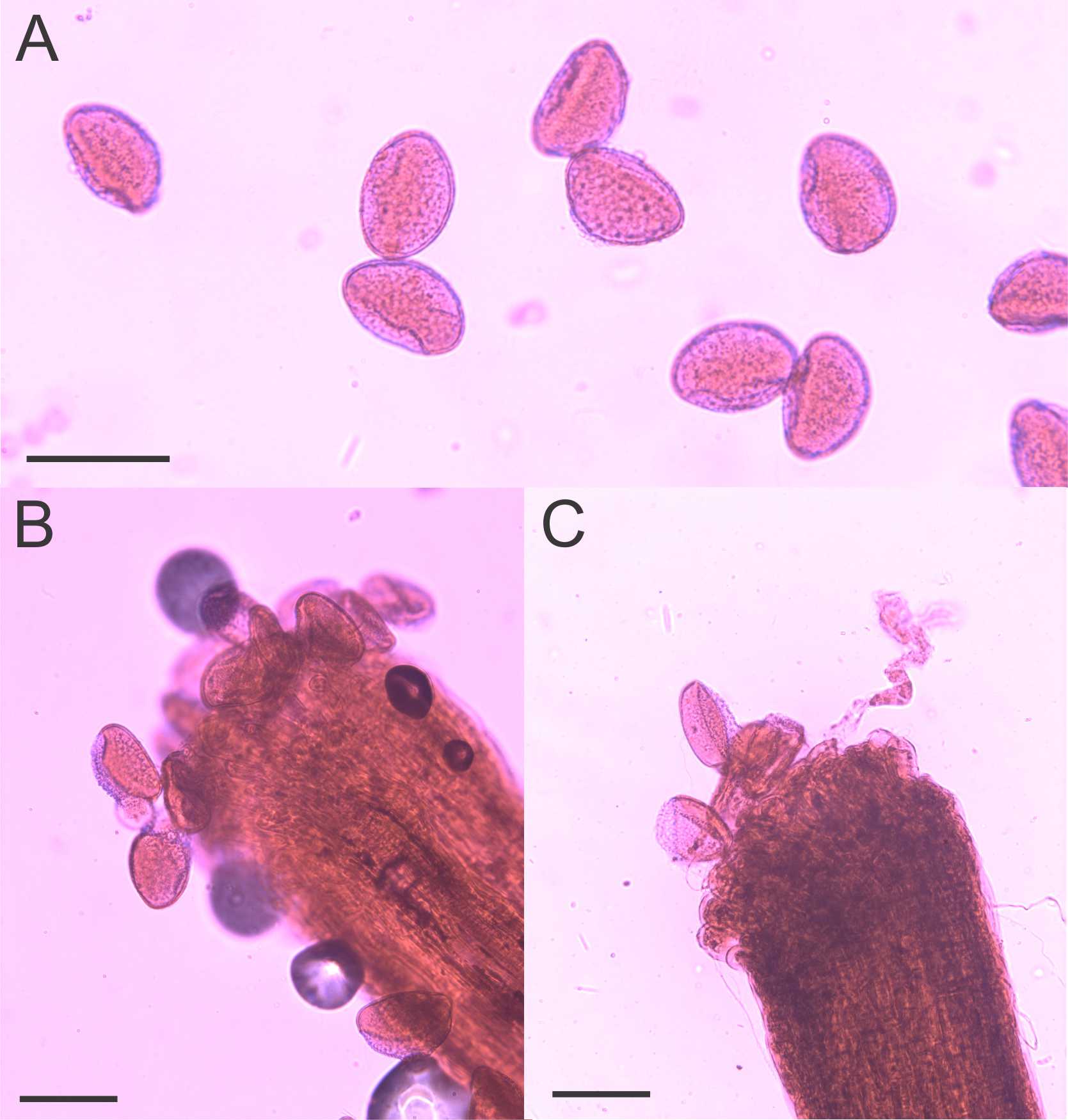


Fig S1. Pollen grains of *Barberetta aurea* in relation to the very small stigma of this species. A. Pollen grains of left-styled morph. B. Pollen on stigma of right-styled morph with pollen. C. Pollen on stigma of left-styled morph. Scale bars = 50 µm
